# Aristolochic acid mutational signature defines the low-risk subtype in upper tract urothelial carcinoma

**DOI:** 10.7150/thno.43251

**Published:** 2020-03-04

**Authors:** Huan Lu, Yuan Liang, Bao Guan, Yue Shi, Yanqing Gong, Juan Li, Wenwen Kong, Jin Liu, Dong Fang, Libo Liu, Qun He, Muhammad Shakeel, Xuesong Li, Liqun Zhou, Weimin Ci

**Affiliations:** 1 Key Laboratory of Genomics and Precision Medicine, Beijing Institute of Genomics, Chinese Academy of Sciences, Beijing 100101, China.; 2 Department of Urology, Peking University First Hospital, Beijing 100034, China.; 3 University of Chinese Academy of Sciences, Beijing 100049, China.; 4 Institute of Urology, Peking University, Beijing 100034, China.; 5 National Urological Cancer Center, Beijing, 100034, China.; 6 Department of Urology, The Third Hospital of Hebei Medical University, Hebei 050051, China.; 7 Jamil-ur-Rahman Center for Genome Research, PCMD, ICCBS, University of Karachi, Pakistan.; 8 Institute of Stem cell and Regeneration, Chinese Academy of Sciences, Beijing 100101, China.

**Keywords:** upper tract urothelial carcinoma, whole-genome sequencing, mutational signature, aristolochic acids, clinical outcome

## Abstract

**Rationale**: Dietary exposure to aristolochic acids and similar compounds (collectively, AA) is a significant risk factor for nephropathy and subsequent upper tract urothelial carcinoma (UTUC). East Asian populations, who have a high prevalence of UTUC, have an unusual genome-wide AA-induced mutational pattern (COSMIC signature 22). Integrating mutational signature analysis with clinicopathological information may demonstrate great potential for risk ranking this UTUC subtype.

**Methods**: We performed whole-genome sequencing (WGS) on 90 UTUC Chinese patients to extract mutational signatures. Genome sequencing data for urinary cell-free DNA from 26 UTUC patients were utilized to noninvasively identify the mutational signatures. Genome sequencing for primary tumors on 8 out of 26 patients was also performed. Metastasis-free survival (MFS) and cancer-specific survival (CSS) were measured using Kaplan-Meier methods.

**Results**: Data analysis showed that a substantial proportion of patients harbored the AA mutational signature and were associated with AA-containing herbal drug intake, female gender, poor renal function, and multifocality. Field cancerization was found to partially contribute to multifocality. Nevertheless, AA Sig subtype UTUC patients exhibited favorable outcomes of CSS and MFS compared to the No-AA Sig subtype. Additionally, AA Sig subtype patients showed a higher tumor mutation burden, higher numbers of predicted neoantigens, and infiltrating lymphocytes, suggesting the potential for immunotherapy. We also confirmed the AA signature in AA-treated human renal tubular HK-2 cells. Notably, the AA subtype could be ascertained using a clinically applicable sequencing strategy (low coverage) in both primary tumors and urinary cell-free DNA as a basis for therapy selection.

**Conclusion**: The AA mutational signature as a screening tool defines low-risk UTUC with therapeutic relevance. The AA mutational signature, as a molecular prognostic marker using either ureteroscopy and/or urinary cell-free DNA, is especially useful for diagnostic uncertainty when kidney-sparing treatment and/or immune checkpoint inhibitor therapy were considered.

## Introduction

Approximately 90-95% of urothelial carcinoma (UC) occurs as urothelial carcinoma of the bladder (UCB), with upper tract urothelial carcinoma (UTUC) accounting for 5-10% 1. Although they have a similar histopathologic appearance, UTUC is a distinct clinical entity with an aggressive clinical behavior and a more advanced presentation compared to UCB 2. East Asian regions, such as China, have a much higher UTUC prevalence, accounting for more than 30% of UCs 3-5. Conceivably, lifestyle behaviors in risk factors for UTUC, such as aristolochic acid (AA)-containing herb drug consumption, may account for the observation 3,6-8. It has been shown that the AA-induced mutational signature is characterized by A:T to T:A transversions 8,9. The unusual genome-wide AA signature, termed signature 22 in COSMIC, holds great potential as “molecular fingerprints” for AA exposure in UTUC 8 and multiple cancer types 7,9.

Clinical interest in the AA-related subtype is increasing, especially in East Asia, since AA- associated UTUCs are prevalent 3,10. Integrating mutational signature analysis with clinicopathological data may be a crucial step toward personalized treatment strategies for this UTUC subtype. However, due to the lack of WGS data and incomplete clinicopathological data in East Asian UTUC patients, the clinical significance of AA-related subtype UTUC remains largely unknown. In this study, we performed WGS on tumor tissues of 90 Chinese UTUC patients. Using the AA mutational signature as a screening tool, we were able to identify the AA Sig subtype of patients who should be stratified into a low-risk group, although with a high incidence of multifocality. As tumor stage is difficult to assert clinically in UTUC, this signature will identify those diagnostic uncertainties when kidney-sparing treatment and/or immune blockade therapy is considered.

## Results

### Genomic characterization of Chinese upper tract urothelial carcinoma

We sequenced 90 UTUC samples (Figure S1) using WGS (~30X) and identified a median of 19,639 (interquartile range (IQR), 16,578 to 32,937) SNVs and a median of 2,197 (IQR, 1,615 to 2,650) indels. A median of 437 (IQR, 355 to 584) coding mutations was noted (Figure S2A). Combined with the clinical information, we found that the increased mutation load was consistent with the prevalence of exposure to potent mutagen AAs (Figure S2B). Next, we examined the candidate driver genes with MutSigCV (Q<0.05). Only two genes, TP53 and FRG2C, were identified (Figure S2C). However, many genes listed in the Cancer Gene Census as known driver genes were affected by nonsilent mutations, including genes that are frequently mutated in Western UTUC patients 11,12, such as KMT2A, C and D (27%) and ARID1A (14%) (Figure S2C). Consistent with a previous study 13, hotspot driver mutations in the promoter of TERT (22% of all the patients) and FGFR3 mutations (2%) were identified in our cohort but at a much lower frequency (Figure S2C). Overall, the landscape of point alterations in our UTUC cohort and Western UTUC cohort was similar, but the prevalence of mutations differed.

### AA mutational signature defines an etiologically distinct subgroup with favorable outcomes

**Figure 1:**
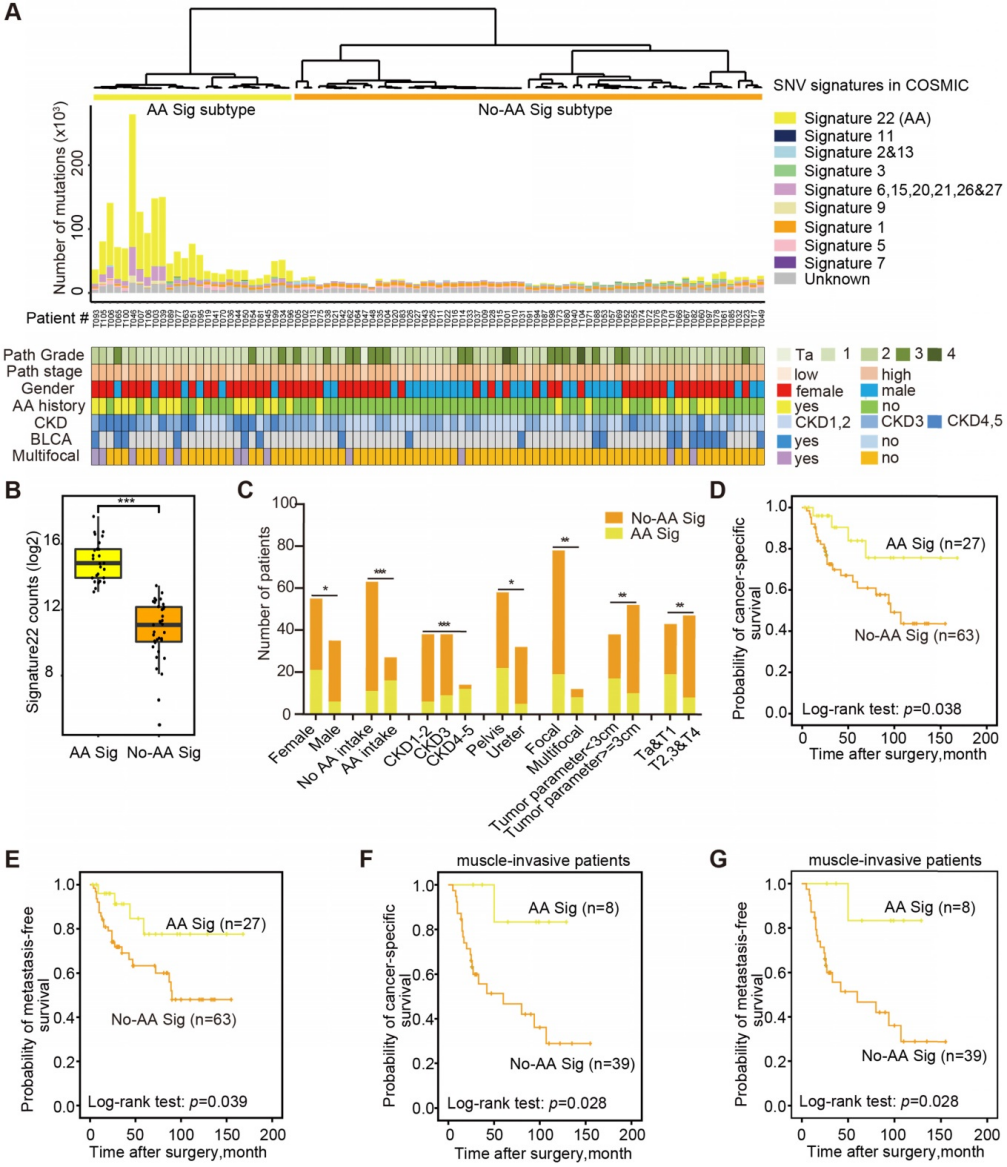
** The AA mutational signature defines etiologically distinct subgroups with favorable outcomes.** (**A**) Bar plot of the number of SNVs attributable to 10 merged signatures in each of the 90 tumors, sorted by hierarchical clustering (dendrogram at top), revealing AA Signature-related (AA Sig, yellow) and non-AA Signature-related (No-AA Sig, orange). Selected clinical features are represented in the bottom tracks. Frozen samples are labeled as T001-T049 and FFPE samples are labeled as T050-T106. (**B**) The box plot shows the mutation counts of signature 22 mutations in tumors within each subtype. Statistical significance was determined by the Wilcoxon rank test, ***P< 0.001. (**C**) The bar graph shows the association between the two subtypes and clinicopathologic features. Statistical significance was determined by the Kruskal-Wallis test, *P<0.05; **P<0.01; ***P< 0.001.** (D)-(G)**, Kaplan-Meier survival curves showed that the mutational signature subtypes can predict both CSS and MFS for the whole cohort, as well as for muscle-invasive UTUC patients. CSS: cancer-specific survival. MFS: metastasis-free survival.* P*-values were calculated by the log-rank test. *n*, the number of cases.

Next, we explored the dynamic interplay of risk factors and cellular processes using mutational signature analysis. We identified 23 mutational signatures defined by COSMIC in our cohort by MutationalPatterns 14. Hierarchical clustering based on the number of SNVs attributable to each signature confirmed two major subtypes: AA Sig and No-AA Sig (Figure 1A). There was a significantly higher number of signature 22 mutations in the AA Sig subtype than in the No-AA Sig subtype (Figure 1B, Wilcoxon rank test, *p*<0.001). Consistent with previous epidemiological studies in Asian patients 6,15-17, we found that the AA Sig subtype was significantly associated with AA-containing herb drug intake, poor renal function, female sex, multifocality and lower T stage (Figure 1C, Table 1). In addition, a Kaplan-Meier plot revealed that the AA Sig subtype exhibited favorable outcomes compared with the No-AA Sig subtype in both cancer-specific survival (CSS) (Figure 1D, log-rank, *p*=0.038) and metastasis-free survival (MFS) (Figure 1E, log-rank, *p*=0.039). Consistently, the AA Sig subtypes also exhibited favorable outcomes in muscle-invasive UTUC patients (log-rank, *p*=0.028 for CSS, log-rank, *p*=0.028 for MFS) (Figure 1F-G). Therefore, the AA mutational signature defines an etiologically distinct subgroup with favorable outcomes.

### Field cancerization may contribute to multifocality in the AA Sig subtype

**Figure 2:**
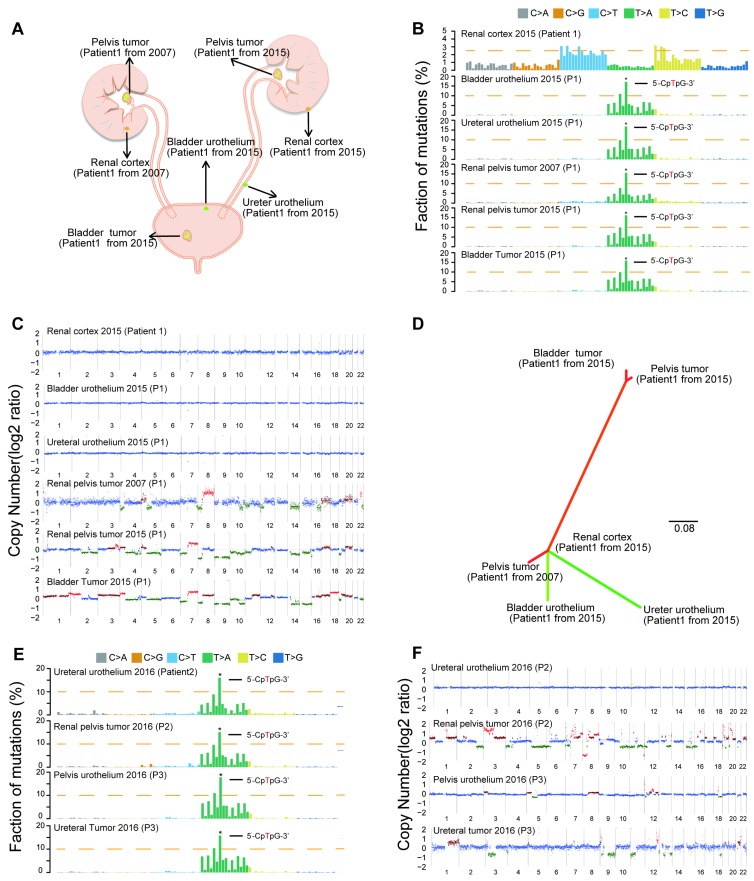
** Field cancerization may contribute to malignant transformation, especially for the AA Sig subtype.** (**A**) Spatial locations of core biopsies of the multifocal AA patient. (**B**) Trinucleotide contexts for somatic mutations in biopsies from the multifocal patient of the AA Sig subtype. (**C**) Copy number plots of the core biopsies from the multifocal patient. (**D**) Phylogenetic relationships of the six samples from the multifocal patient were deciphered using mrbayes_3.2.2. Branch lengths are proportional to the number of somatic mutations separating the branching points. (**E**) Trinucleotide contexts for somatic mutations in biopsies of another two AA Sig subtype patients. (**F**) Copy number profiles of another two AA Sig subtype patients.

Consistent with our previous epidemiological study 17, we found an increased rate of multifocality and high bladder recurrence in the AA Sig subtype (Figure S2B, Table 1). Field cancerization 18, which is the development of a field with genetically altered cells, has been proposed to explain the development of multiple primary tumors and local recurrence. Therefore, we further sequenced three AA Sig subtype patients, including a multifocal patient (Figure S3 and Table S1). We did find similar numbers of SNVs and indels in the morphologically normal urothelium specimens in the multifocal patient (Table S2). Strikingly, the AA mutational signature was consistently identified in urothelium specimens and tumor tissues in multifocal patients (Figure 2A-B), which indicated that AA exposure may contribute to field cancerization. However, copy number alterations were not identified in the urothelium tissues of this patient (Figure 2C). Moreover, in the multifocal patient, the urothelial tumor in the renal pelvis from 2007 shared no genetic alterations with a renal pelvis tumor from 2015 or a bladder tumor from 2015. However, the two tumors from 2015 were genetically related (Figure 2D). Therefore, field cancerization and intraluminal seeding could co-contribute to multifocality and increased bladder recurrence in AA Sig subtype patients. The AA mutational signature was consistently identified in urothelium specimens and tumor tissues in the other two AA Sig subtype patients (Figure 2E). Strikingly, we found copy number alterations in urothelium specimens of an AA Sig subtype patient (Figure 2F). Similarly, the copy number alteration pattern in urothelium specimens was not consistent with the matched tumor, which suggested that AA may also induce copy number alterations in the morphologically normal urothelium.

### Potential for immunotherapy in the AA Sig subtype of UTUC patients

**Figure 3:**
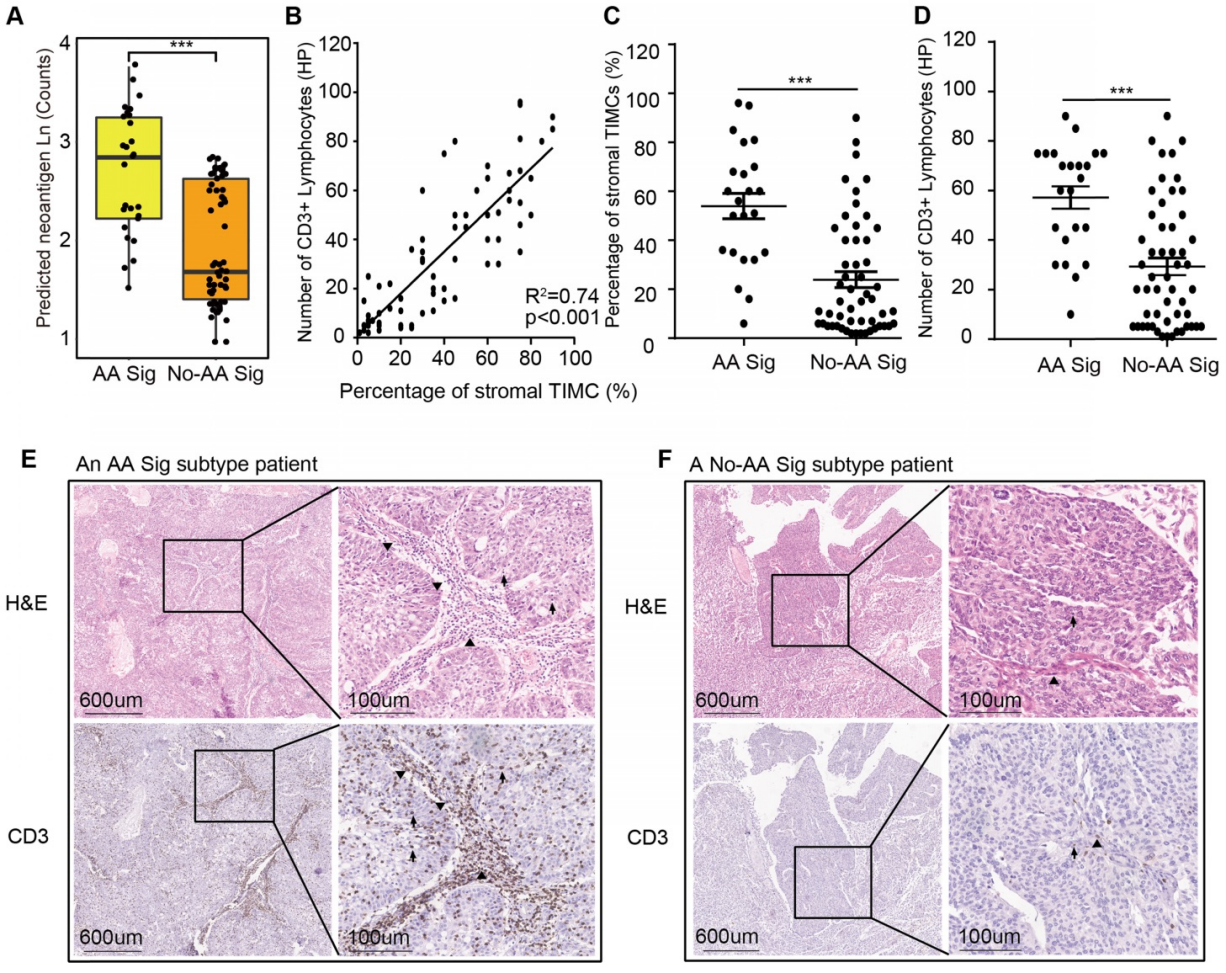
** High neoantigen burden and heavy tumor-infiltrating lymphocytes in the AA Sig subtype.** (**A**) Neoantigen burden was significantly higher in the AA group. Statistical significance was determined by the Wilcoxon rank test (***P<0.001). (**B**) Positive correlation of the percentage of stromal tumor-infiltrating mononuclear cells (TIMCs) and the number of CD3^+^ lymphocytes in 76 UTUC patients in our cohort (*n*_AA Sig_=23; *n*_No-AA Sig_=53). (**C-D**) The percentages of stromal TIMCs **(C)** and CD3^+^ lymphocytes **(D)** are shown in each subtype of patients. Statistical significance was determined by the Wilcoxon rank test (**P<0.01, ***P<0.001). HP represents a high-power field. **(E-F)** Images of TIMCs and CD3^+^ lymphocytes of a representative patient from the AA Sig subtype **(E)** and the No-AA Sig subtype **(F)** Triangle highlighting the TIMCs or CD3^+^ lymphocytes in the stromal tumour region. The arrow highlights the TIMCs or CD3^+^ lymphocytes in the intratumor region.

The AA Sig subtype bears high mutation burdens and thus may be a good candidate for immune checkpoint blockade therapy 19. We predicted neoantigens binding to patient-specific human leukocyte antigen (HLA) types. The AA Sig subtype had the highest number of predicted neoantigens (Figure 3A). Moreover, it has been reported that lymphocyte infiltration, especially CD3^+^ lymphocytes, in the tumor region is associated with improved survival in a range of cancers, including urothelial cancer 20-22, and the number of tumor- infiltrating lymphocytes independently correlates with progression-free survival in non-small-cell lung carcinoma patients treated with nivolumab immunotherapy. Therefore, we further evaluated the extent of tumor-infiltrating mononuclear cells (TIMCs) and CD3^+^ lymphocytes in 76 available samples (Table S3). We found that the number of CD3^+^ lymphocytes was positively associated with the number of stromal TIMCs (*R*^2^=0.74; *p*<0.001) (Figure 3B). The AA Sig subtype had higher numbers of both stromal TIMCs (Wilcoxon rank test, *p*<0.001) and CD3^+^ lymphocytes (Wilcoxon rank, *p*<0.001) (Figure 3C-D). Representative images from a patient with the AA Sig subtype and the No-AA Sig subtype are shown in Figure 3E and 3F, respectively.

### AA mutational signature as “molecular fingerprint” for inferring previous AA exposure and AA Sig subtype patients by urinary cell-free DNA

**Figure 4:**
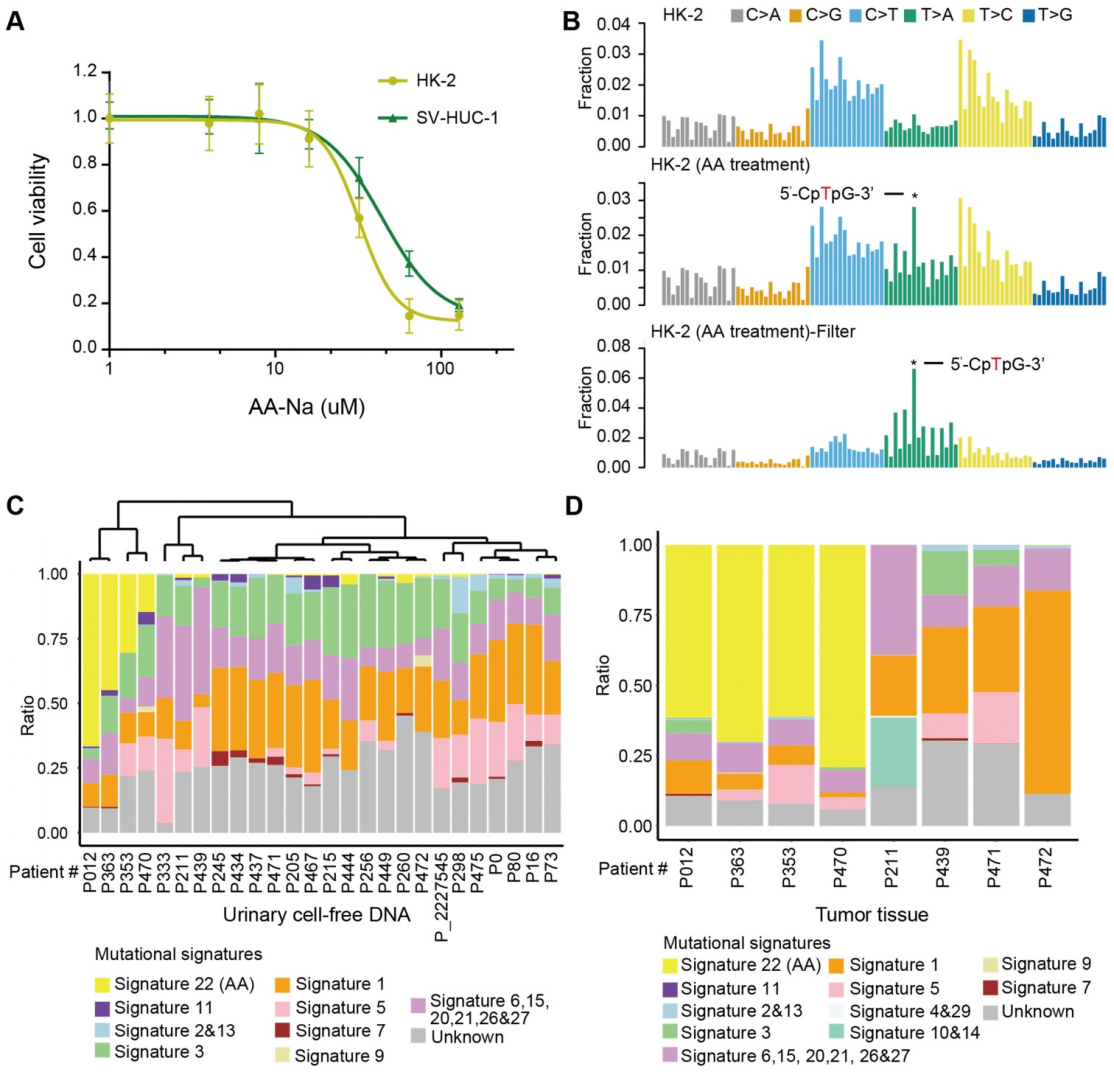
** AA mutational signature as “molecular fingerprints” for inferring previous AA exposure and AA Sig subtype patients by urinary cell-free DNA** (**A**) The AA killing curve of the HK-2 and SV-HUC-1 cells. (**B**) Trinucleotide contexts for mutations in HK-2 cells and AA-treated HK-2 cells. The mutations in AA-treated HK-2 cells were further filtered by untreated HK-2 cells. Trinucleotide contexts for the filtered mutations in AA-treated HK-2 cells are shown in the bottom panel. **(C)** The box plot shows the ratio of 10 merged signatures in the cell-free DNA at low coverage. **(D)** The box plot shows the ratio of 12 merged signatures in the selected primary tumors of matched urinary cell-free DNA samples.

First, we experimentally verified that AA alone, as a purified isolated compound, was sufficient to cause AA mutational signatures in human renal tubular cell HK-2 and human uroepithelium cell SV-HUC-1. We found that HK-2 cells (IC50 31.97 µM) were more sensitive to AA treatment than SV-HUC-1 cells (IC50 43.78 µM) (Figure 4A). Next, we treated HK-2 cells (4.5 months) and SV-HUC-1 cells (3 months) with AA at 50% IC50, which resulted in the development of mixed clones. Consistent with previous studies in several types of cells 7,23,24, we identified AA mutational signature mutations in HK-2 cells (Figure 4B). The proportion of the AA mutational signature in HK-2 cells was more evident when called mutations were filtered by untreated cells (Figure 4B). However, SV-HUC-1 cells hardly proliferated following AA exposure, and the AA mutational signature was not identified (Figure S4A). Copy number changes were also identified in AA-treated HK-2 cells but not in SV-HUC-1 cells (Figure S4B). Further mechanism study of AA exposure in SV-HUC-1 cells should maintain cells in culture with occasional passaging until cultures emerged from senescence as previously reported in human primary murine embryonic fibroblasts 23,24.

Moreover, we identified an AA mutational signature in histologically “normal” urothelial cells. In a published study 25, a similar “field effect” was also identified in a Chinese UTUC patient. Thus, we evaluated whether we can take advantage of this “field effect” and used a noninvasive urine test to screen AA Sig subtype patients in urine sediment and/or cell-free DNA. One practical question that arises is how this approach could be implemented clinically. It has been shown that successful detection of the AA signature in urothelial tumors using archived FFPE specimens and low-coverage exome sequencing 26. Thus, using our published low-coverage WGS data 27 for urinary cell-free DNA from 26 patients with UTUC, we detected a large proportion of the AA mutational signature (nearly >15%) in 4 out of 26 patients (Figure 4C). To further validate whether the AA mutational signature in urinary cell-free DNA inferred the AA Sig subtype patients, we performed whole-genome sequencing in primary tumors of 8 urinary cell-free DNA matched patients: 4 with a high proportion (nearly >15%) and 4 with a very low proportion (<5%) of AA mutational signatures in cell-free DNA. Consistently, high proportion (>50%) of AA signature mutations was identified in matched tumor tissues in all 4 patients with high proportion (nearly >15%) of AA signature mutations in cfDNA but not in any of the 4 patients with low proportion (<5%) of AA signature mutations in cfDNA (Figure 4D). Taken together, we confirmed that the AA mutational signature in cell-free DNA can infer the AA Sig subtype patients.

## Discussion

In this study, the comprehensive genomic analysis of Chinese UTUC patients shows a significant association of the AA mutational signature with the consumption of AA-containing herbal formulations. Fifty-nine percent of patients (16/ 27) with a self-reported AA intake history of over 1 year were classified as AA Sig subtype, while there are also 17% (11/63) of patients without self-reported AA intake history which were classified as AA Sig subtype (Table 1, Supplementary file 1). This finding may be due to the difficulty of tracking the dosage of AAs from various herbal remedies. In our study, only 70 AA-containing single products and mixed herbal formulas were considered (Table S4). Nevertheless, AA-containing herbs should be discouraged for clinical use due to their nephrotoxic and UTUC-promoting potential.

Notably, our AA Sig subtype patients presented favorable clinical outcomes. This finding is contrary to previous reports in which the AA Sig subtype was significantly associated with multifocality and impaired renal function. According to the latest EAU guidelines, multifocality as a preoperative prognostic factor would define UTUC patients as high-risk, and radical nephroureterectomy is the standard intervention for such patients 1. Very few AA Sig subtype patients can receive chemotherapy due to aggravation of renal function after radical surgery. AA Sig subtype patients normally experience multiple surgeries and eventually bilateral nephrectomy, such as the multifocal patient of the present study (one side radical nephroureterectomy in 2007 and the other side in 2015). However, in the AA Sig subtype being a low-risk group, kidney-sparing surgical management and close and stringent follow-up may be recommended. Furthermore, advanced and/or metastatic disease may be subjected to immune checkpoint blockade therapy. More importantly, we found that even though fewer SNVs were called with low-coverage WGS data (Figure S5A), the proportion of the AA mutational signature in backgrounds of other mutational signatures can be successfully retrieved by low-coverage WGS data (Figure S5B). Therefore, using the AA mutational signature as a screening tool by low-coverage WGS data with either diagnostic ureteroscopy or urinary cell-free DNA has great clinical significance for disease management, although this needs further investigation in clinical practice using a larger cohort. Additionally, the AA mutational signature has also been identified in several other types of cancer patients from both East Asia and Europe, such as kidney cancer and liver cancer 7,9,28,29. The AA mutational signature may serve as a secondary prevention tool by screening for AA-associated cancers or for kidney disease in patients suspected or known to be exposed to AA.

Collectively, our study provides the most comprehensive genomic profile of Chinese UTUC patients to date. Use of the AA mutational signature as a screening tool may accelerate the development of novel prognostic markers and personalized therapeutic approaches for AA Sig subtype UTUC patients.

## Methods and Materials

### Patient cohort

All fresh UTUC samples in this study were obtained from Peking University First Hospital (Grant No.2015(977)). These fresh samples were stored in liquid nitrogen immediately after surgery. Formalin-fixed, paraffin-embedded (FFPE) UTUC samples were provided by the Institute of Urology after pathologic diagnosis. The main endpoint events consisted of cancer-specific survival (CSS) and metastasis-free survival (MFS). All patients were not treated with neoadjuvant chemotherapy. The study was approved by the Ethics Committee of Peking University First Hospital.

### AA exposure assessment

AA exposure assessment was performed according to self-reported data on 70 AA and its derivative-containing herb drug (collectively, AA) intake 17,30. These herbs were taken as single products (Guan Mu Tong (Aristolochia manshuriensis Kom), Guang Fangchi (Aristolochia fangchi), Qing Mu Xiang (Radix Aristolochiae), Ma Dou Ling (Fructus Aristolochiae), Tian Xian Teng (Caulis Aristolochiae), Xun Gu Feng (herba Aristolochiae mollissimae), and Zhu Sha Lian (Aristolochia cinnabarina)) or were components of mixed herbal formulas (e.g., Guan Mu Tong in the Long Dan Xie Gan mixture). The accumulated self-reported usage of the above drugs for more than a year was termed AA exposure patients. Clinical and demographic information was obtained from a prospectively maintained institutional database.

### Whole-genome sequencing

For whole-genome sequencing, genomic DNA from FFPE cancer samples was isolated using the Quick-DNA™ FFPE Kit and Genomic DNA Clean & Concentrator (Zymo Research, CA, US) and from fresh cancer tissue samples using the QIAmp DNA Mini Kit (QIAGEN Inc., MD, US). The DNA sequencing libraries were prepared using the NEBNext Ultra II DNA Library Prep Kit for Illumina (New England Biolabs, MA, US) following the manufacturer's instructions. Briefly, the genomic DNA was fragmented using a Covaris S2 Ultrasonicator instrument (Covaris Inc., MA, US). The sheared DNA was repaired and 3' dA-tailed using the NEBNext Ultra II End Repair/dA-Tailing Module unit and then ligated to paired-end adaptors using the NEBNext Ultra II Ligation Module unit. After purification by AMPure XP beads, the DNA fragments were amplified by PCR for 6-8 cycles. The quality of the DNA sequencing library was assessed with a Bioanalyzer 2100 system. Finally, the libraries were pooled and sequenced with the HiSeq X Ten platform (Illumina, San Diego, CA, US) according to the manufacturer's instructions, generating 2 x 150-bp paired-end reads.

### Single nucleotide variations and insertions/deletions calling

The quality of short DNA reads was controlled by Trimmomatic 31. The good quality PE reads were aligned with the human reference genome hg19 (http://genome.ucsc.edu/) using the BWA-MEM tool 32. The reads mapped with the reference at the same coordinates were removed using Picard. Furthermore, realignment at insertion/deletion sites (indels) and base quality score recalibration (BQSR) were performed following the best practices of the GATK pipeline 33. Single nucleotide variations (SNVs) and indels were called using VarScan2 34 and Vardict 35, and then Rtg tools 36 was used to remove variants called in a set of 1000 healthy Chinese individuals 37 and obtain the common variants called by the two software packages. Further filtering criteria were carried out according to reference 38,39. SNVs meet these criteria were removed: 1) The median shortest distance of the variant position within the read to either aligned end is less than 10; 2) The median absolute deviation of the shortest distance of the variant position within the read to either aligned end is less than 3; 3) The proportion of reads at the variant position with low mapping quality (less than 1) is greater than 10%; 4) The median mapping quality of variant reads is less than 40; 5) The median base quality at the variant position of variant reads is less than 20; 6) The strand bias for variant reads covering the variant position, i.e. the fraction of reads in either direction, is less than 0.02,unless the strand bias for all reads is also less than 0.2; 7) The length of repetitive sequence adjacent to the variant position, where repeats can be 1-, 2-, 3-, or 4-mers, is 12 or more; and 8) The largest number of variant positions within any 50 base pair window surrounding, but excluding, the variant position is greater than 2. Then, we set the mutational allele frequency cutoff as more than 0.25 and less than 0.75 according to the allele frequency distribution of the samples. (SNVs with allele frequency more than 0.75 were more likely germline mutations and SNVs with allele frequency less than 0.25 were more enriched in FFPE samples). Finally, Annovar 40 was applied to remove variants whose mutation frequency was no less than 0.001 in the 1000 Genomes project phase 3, latest Exome Aggregation Consortium (ExAC) dataset, NHLBI-ESP project with 6500 exomes, latest Haplotype Reference Consortium database, and latest Kaviar database. SNVs which are associated with Pathogenic in clinvar or annotated with urinary associated in cosmic were retrieved. When calling SNVs from low-coverage data, we did not filter the SNVs based on allele frequency. Phylogenetic relationships of the six samples from the multifocal patient were deciphered with SNVs using mrbayes_3.2.2 41.

### Mutational signature analysis

The R (3.5.1) package MutationalPatterns 14 was used to determine mutational signatures in each sample by using “fit_to_signatures” following the authors' guidelines. The output files of MutationalPatterns are presented in Supplementary file 2 and 3. We discovered 23 COSMIC signatures that were merged by shared etiologies into 10 signatures in our cohort. We named signature 22 as AA. Hierarchical clustering was performed by the number of SNVs attributable to each signature 42.

### Neoantigen prediction

HLAscan 43 was used to genotype the HLA region with HLA-A, HLA-B and HLA-C taken into consideration (default parameters). NetMHC4.0 44 was used for predictions of peptide-MHC class I interactions. Nonsynonymous SNVs were used to perform this analysis. An in-house script was used to obtain possible 9-amino acid sequences covering the mutated amino acids according to the manual. We counted the strong binders (%rank < 0.5) according to the manual of NetMHC4.0.

### Assessing tumor-infiltrating lymphocytes

Tumor-infiltrating mononuclear lymphocytes were measured according to a standardized method from the International Immuno-Oncology Biomarkers Working Group 45. To evaluate the CD3^+^ lymphocytes in tumor sections, anti-CD3 antibody (ab5690, 1:10000; Abcam) was used in a histochemical assay.

### Statistical analysis

The variables of different groups were compared using the Wilcoxon rank test and Kruskal-Wallis test as indicated. Kaplan-Meier (K-M) analysis was used to evaluate the associations of the classifiers with metastasis-free survival (MFS) and cancer-specific survival (CSS). Two-sided *P*-values < 0.05 were considered statistically significant. All statistical analyses were conducted using SPSS version 20.0 (IBM Corporation, America) and R (3.5.1).

### Cell lines

The human normal renal tubular epithelial cell line HK-2 was purchased from American Type Culture Collection (ATCC, Manassas, VA, US). Referring to prior methods 7,46, HK-2 cells were cultured in RPMI-1640 (HyClone, Logan, UT) medium with 10% fetal calf serum (HyClone Laboratories Inc., Logan, UT). Human bladder epithelial permanent SV-HUC-1 cells were purchased from American Type Culture Collection (ATCC, Manassas, VA). The SV-HUC-1 cells were cultured in F-12K (GIBCO) medium with 10% fetal calf serum (HyClone Laboratories Inc., Logan, UT). Both cell lines were treated with aristolochic acid I sodium salt (A9451, SIGMA, US) at 50% IC50: HK-2 (15.98 µM) for four and half months and SV-HUC-1 (21.89 µM) for three months.

### Study approval

The study was approved by the Ethics Committee of Peking University First Hospital.

### Data availability

The raw sequence data reported in this paper have been deposited in the Genome Sequence Archive 47 in BIG Data Center 48, Beijing Institute of Genomics (BIG), Chinese Academy of Sciences (accession number HRA000029 and GVM000054 (GVM)). That can be accessed at http://bigd.big.ac. cn/gsa-human/s/HiObV4f3 and https://bigd.big.ac. cn/gvm/getProjectDetail?project=GVM000054.

## Supplementary Material

Supplementary figures, tables, and file legends.

Supplementary file 1.

Supplementary file 2.

Supplementary file 3.

## Figures and Tables

**Table 1:** Clinical characteristics stratified by mutational signature

Variable	No. (%)	AA Sig (%)	No-AA Sig (%)	*P*-values
**Total**	90	27	63	
**Age**				0.355
<65 y	40(44.4)	14(51.8)	26(41.3)	
≥65 y	50(55.6)	13(48.2)	37(58.7)	
**Smoking**				0.092
Absent	74(82.2)	25(92.6)	49(77.8)	
Present	16(17.8)	2(7.4)	14(22.2)	
**AA intake**				**<0.001**
Absent	63(70.0)	11(40.7)	52(82.5)	
Present	27(30.0)	16(59.3)	11(17.5)	
**Sex**				**0.034**
Female	55(61.1)	21(77.8)	34(54.0)	
Male	35(38.9)	6(22.2)	29(46.0)	
**CKD**				**<0.001**
1~2	38(42.2)	6(22.2)	32(50.8)	
3	38(42.2)	9(33.3)	29(46.0)	
4~5	14(15.6)	12(44.4)	2(3.2)	
**Primary tumor location**				**0.027**
Pelvis	58(64.4)	22(81.5)	36(57.1)	
Ureter	32(35.6)	5(18.5)	27(42.9)	
**Multifocality**				**0.003**
Absent	78(86.7)	19(70.4)	59(93.7)	
Present	12(13.3)	8(29.6)	4(6.3)	
**Tumor size**				**0.009**
<3 cm	38(42.2)	17(63.0)	21(33.3)	
≥3 cm	52(57.8)	10(37.0)	42(66.7)	
**Architecture**				0.361
Papillary	64(71.1)	21(77.8)	43(68.3)	
Sessile	26(28.9)	6(22.2)	20(31.7)	
**T stage**				**0.005**
Ta, 1	43(47.8)	19(70.3)	24(38.1)	
T2, T3&T4	47(52.2)	8(29.6)	39(61.9)	
**Grade**				0.797
Low	25(27.8)	7(25.9)	18(28.6)	
High	65(72.2)	20(74.1)	45(71.4)	
**N stage**				0.098
N0 or Nx	83(92.2)	27(100.0)	56(88.9)	
N1~2	7(7.8)	0(0.0)	7(11.1)	
**Postoperative chemotherapy**				0.053
Absent	81 (90.0)	27 (100.0)	54(85.7)	
Present	9 (10.0)	0 (0.0)	9(14.3)	
**Postoperative radiotherapy**				0.317
Absent	85 (94.4)	27 (100.0)	58(92.1)	
Present	5 (5.6)	0 (0.0)	5(7.9)	

Nx: No lymph node dissection was performed.

## Abbreviations

AA: aristolochic acids
UTUC: upper tract urothelial carcinoma
WGS: whole-genome sequencing
UC: urothelial carcinoma
UCB: urothelial carcinoma of the bladder
IQR: interquartile range
CSS: cancer-specific survival
MFS: metastasis- free survival
HLA: human leukocyte antigen
TIMCs: tumor-infiltrating mononuclear cells
FFPE: formalin-fixed, paraffin-embedded
PE: paired-end
BQSR: base quality score recalibration
SNVs: single nucleotide variants
