# Supplementary material for: Aristolochic acid mutational signature defines the low-risk subtype in upper tract urothelial carcinoma
Source: Theranostics. 2020 Mar 4;10(10):4323–33. doi: 10.7150/thno.43251 (PMC7150494; doi:10.7150/thno.43251)
Supplement: Supplementary file 1 — Supplementary figures, tables, and file legends. [file thnov10p4323s1.pdf]

Supplementary Material

1. Supplemental Figures

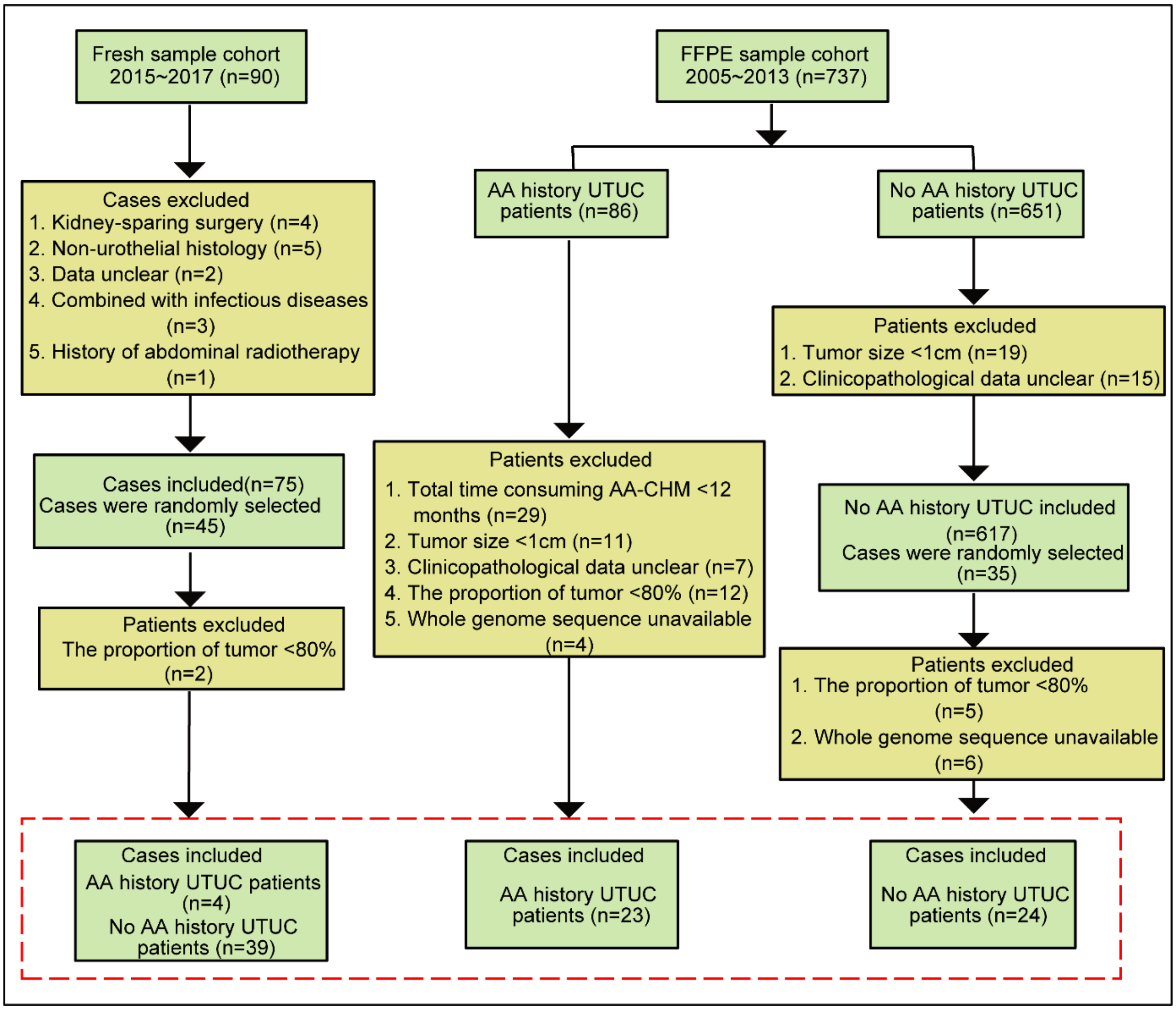

Supplemental Figure S1. Overview of the patient selection. None of patients received neoadjuvant treatment.

RNU: radical nephroureterectomy, AA: aristolochic acid, FFPE: formalin-fixed paraffin-embedded.

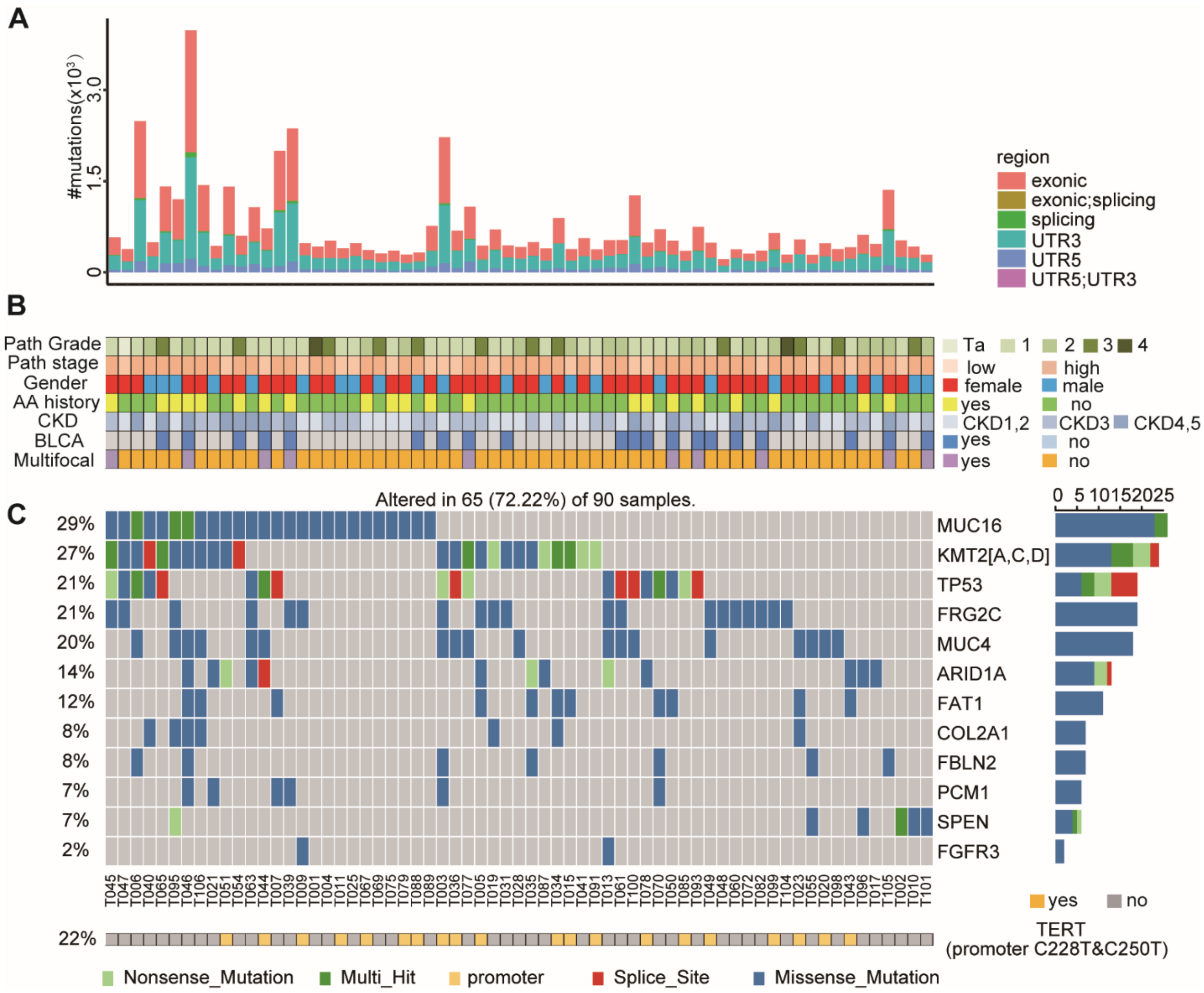

Supplemental Figure S2. The mutational landscape of UTUC. (**A**) Mutation rate and type. (**B**) Selected patient clinical features are included. BLCA: synchronous bladder cancer, bladder recurrence and bladder cancer history. AA: aristolochic acid. CKD: chronic kidney disease. (**C**) Cancer consensus frequently mutated genes and mutation types. Hotspot of driver mutations in promoter of TERT (C228T and C250T).

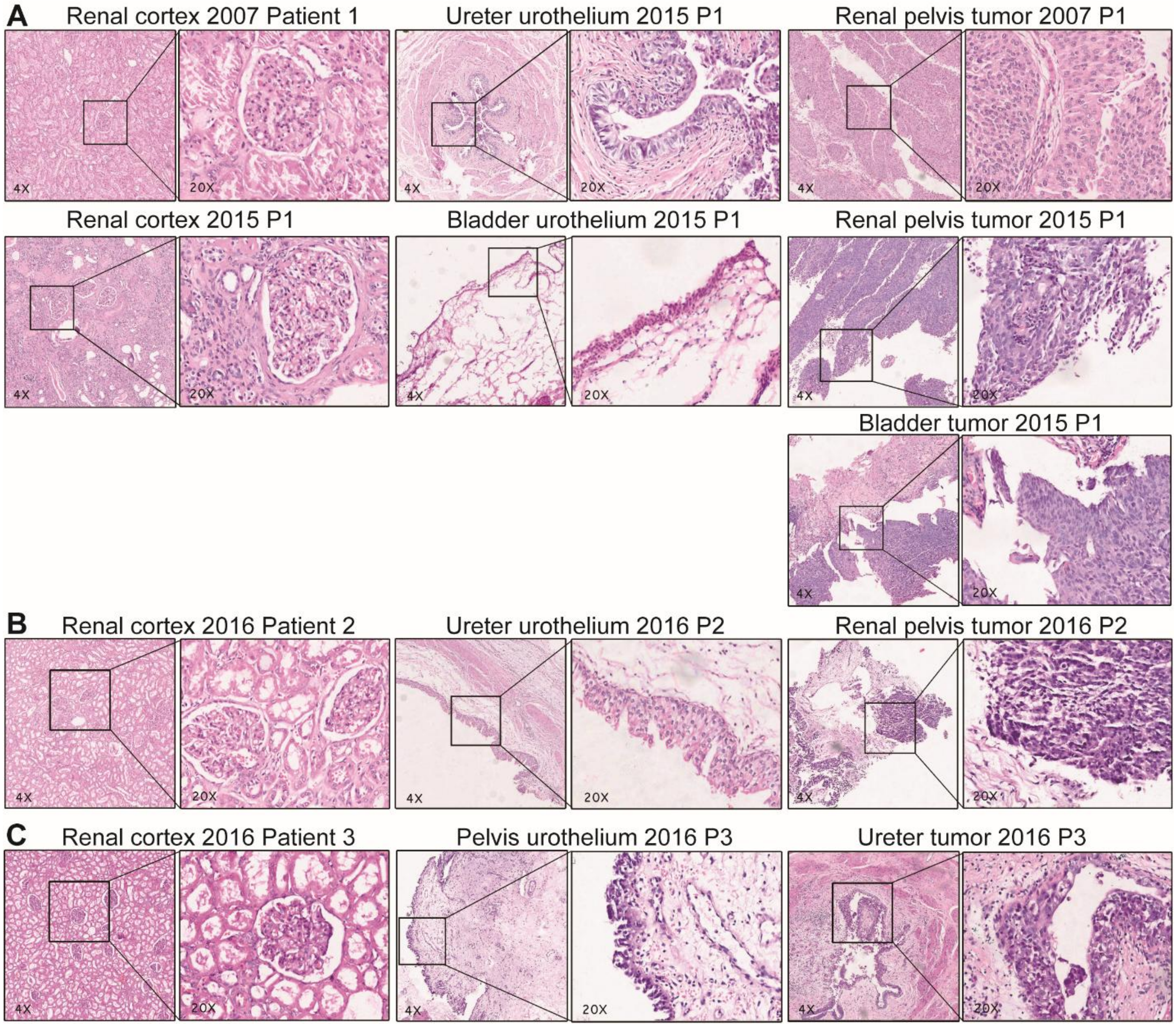

Supplemental Figure S3. Histology of 13 sections from 3 UTUC patients within the AA subtype. (**A-C**) Histological sections in three patients (P1, P2 and P3) identified by H&E staining (4x). Insets represent magnification of the black frame area (20x). RC: renal cortex; PU: pelvis urothelium; PT: renal pelvis tumor; UU: ureter urothelium; UT: ureter tumor; BU: bladder urothelium; BT: bladder tumor. Sample names are in the form of patient ID+sample type. For example, P3PU represents the pelvis urothelium from patient 3.

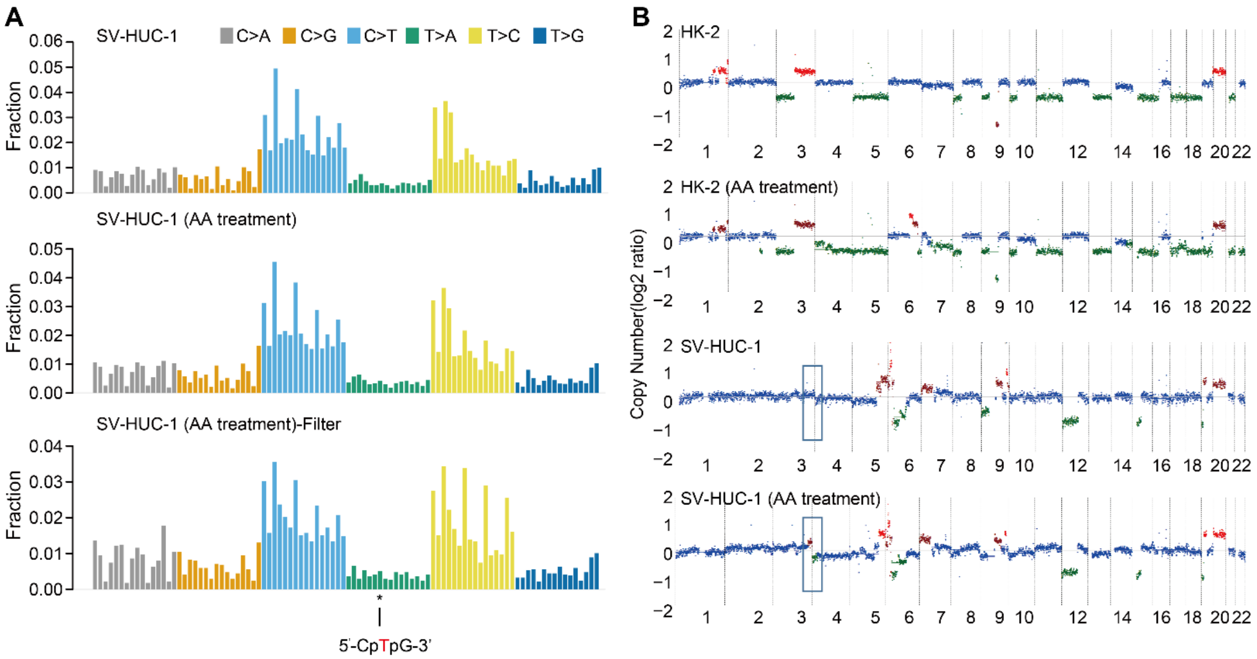

Supplemental Figure S4. **AA mutational signature can be detected in experimentally AA treated human cells.** (**A**) Trinucleotide contexts for mutations in SV-HUC-1 cells and AA-treated SV-HUC-1 cells. The mutations in AA-treated SV-HUC-1 cells are further filtered by untreated SV-HUC-1 cells. Trinucleotide contexts for the filtered mutations in AA-treated SV-HUC-1 cells are shown in bottom panel. (**B**) Copy number changes are identified after AA treatment in HK-2 cells but not SV-HUC-1 cells.

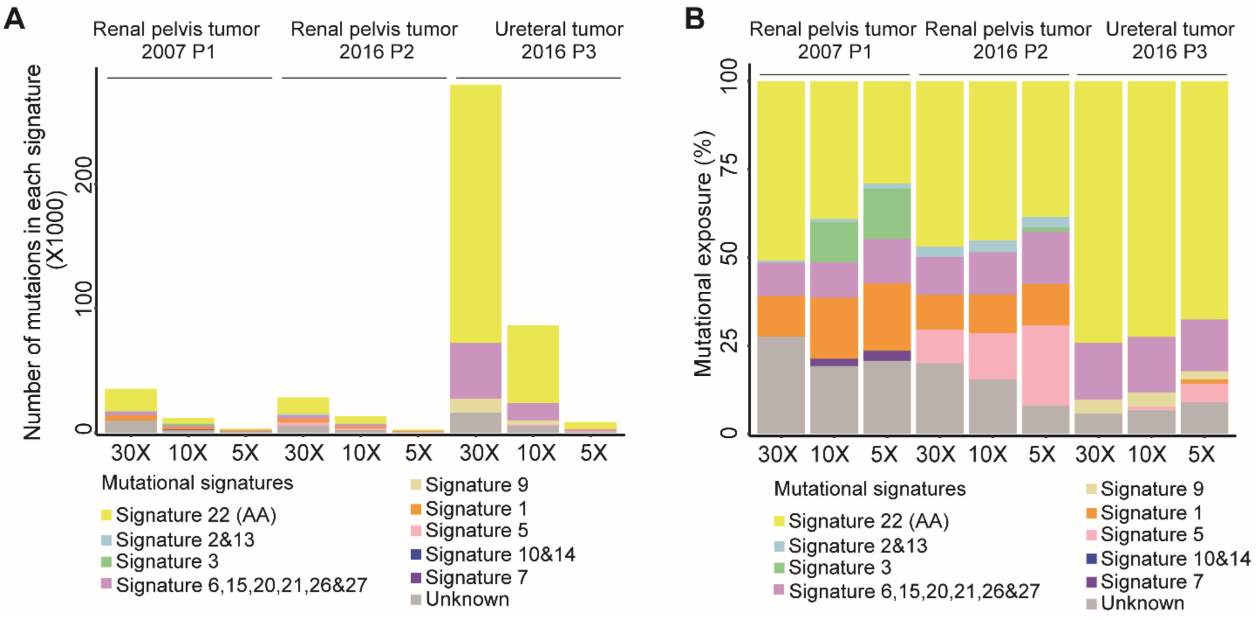

Supplemental Figure S5. Mutational signature box plots at different depth in three patients. (A) The box plot shows the absolute SNV numbers of merged 10 signatures in the three AA tumor tissues calculated at different coverage. (**B)** The box plot shows the ratio of merged 10 signatures in the three AA tumor tissues calculated at different coverage.

2. Supplemental Tables

Supplemental Table S1: The clinicopathological characteristics for the three UTUC patients from the AA subtype.

Patient	Age (yr)	Gender	Germline	Tumor ID	Urothelium ID	Surgery	Grade	pT
**P1**	64	Female	P1RC_1	P1PT1		2007.10	HG	pT3
			P1RC_2	P1PT2	P1UU	2015.05	LG	pT1
				P1BT	P1BU	2015.05	LG	pTa
**P2**	68	Female	P2RC	P2PT	P2UU	2016.08	HG	pT1
**P3**	53	Female	P3RC	P3UT	P3UU	2016.09	HG	pT2

RC= renal cortex; PU= pelvis urothelium; PT = renal pelvis tumor; UU= ureteral urothelium; UT= ureteral tumor; BU= bladder urothelium; BT= bladder tumor; HG = high grade; and LG = low grade.

Supplemental Table S2: Sequencing information and somatic mutation rates for the samples.

Sample	Patient	Average sequencing depth (after removing duplicates)	Mutations (*n*)
Renal cortex 2007 (P1RC1)	P1	32X	
Renal cortex 2015 (P1RC2)	P1	32X	2,451
Bladder urothelium 2015 (P1BU)	P1	32X	183,751
Ureteral urothelium2015 (P1UU)	P1	33X	345,771
Renal pelvis tumor 2007 (P1PT1)	P1	33X	93,300
Renal pelvis tumor 2015 (P1PT2)	P1	30X	493,683
Bladder Tumor 2015 (P1BT)	P1	30X	501,893
Renal cortex 2016 (P2RC)	P2	28X	
Ureteral urothelium 2016 (P2UU)	P2	24X	22,069
Renal pelvis tumor 2016 (P2PT)	P2	36X	63,219
Renal cortex 2016 (P3RC)	P3	29X	
Pelvis urothelium 2016 (P3PU)	P3	31X	414,284
Ureteral Tumor 2016 (P3UT)	P3	31X	725,583

Supplemental Table S3: Proportion of stromal TIMCs and the number of CD3$^+$ lymphocytes in 76 UTUC patients

Variable	Number (%)
**Total**	76
**Stromal of TIMC (%)**	
**Focal (0~10)**	22 (28.9)
**Mild (11~30)**	18 (23.7)
**Moderate (31~60)**	17 (22.4)
**Severe (61~100)**	19 (25.0)
**No. of CD3$^+$ TIMC (high power field)**	
**0~10**	24 (31.6)
**11~30**	16 (21.0)
**31~60**	23 (30.3)
**61~100**	13 (17.1)

Supplemental Table S4: The detail list of AA and/or similar compounds-containing herb drugs surveyed by current study.

#	Drug names(In Mandarin Chinese)	AA and their derivatives (In Mandarin Chinese)	AA and their derivatives containing herbs	Status
1	*Long Dan Xie Gan Wan (watered pill,decoction,granule)*	*Guan Mu Tong*	*Aristolochia manshuriensis Kom*	Replaced by *Akebiae Caulis* since 2003
2	*Guan Mu Tong Tang Ji (decoction)*	*Guan Mu Tong*	*Aristolochia manshuriensis Kom*	Replaced by *Akebiae Caulis* since 2003
3	*Dao Chi Wan (pill)*	*Guan Mu Tong*	*Aristolochia manshuriensis Kom*	Replaced by *Akebiae Caulis* since 2003
4	*Gan Lu Xiao Dou Wan (pill)*	*Guan Mu Tong*	*Aristolochia manshuriensis Kom*	Replaced by *Akebiae Caulis* since 2003
5	*Dou Long Wan (pill)*	*Guan Mu Tong*	*Aristolochia manshuriensis Kom*	Replaced by *Akebiae Caulis* since 2003
6	*Fu Shen Ning Pian (pill)*	*Guang Fang Ji*	*Radix Aristolochiae Fangchi*	Replaced by *Radix Stephaniae Tetrandrae* since 2004
7	*Feng Shi Ling Xian Ye (solution)*	*Guang Fang Ji*	*Radix Aristolochiae Fangchi*	Replacedby *Radix Stephaniae Tetrandrae* since 2004
8	*Fu Fang Xia Tian Wu Pian (pill)*	*Guang Fang Ji*	*Radix Aristolochiae Fangchi*	Replaced by *Radix Stephaniae Tetrandrae* since 2004
9	*Gu Xian Pill (pill)*	*Guang Fang Ji*	*Radix Aristolochiae Fangchi*	Replaced by *Radix Stephaniae Tetrandrae* since 2004
10	*Guan Xin Su He Wan (pill,capsule)*	*Qing Mu Xiang*	*Radix Aristolochiae*	Replaced by *Inulae Radix* since 2004
11	*Shu Gan Li Qi Wan (pill)*	*Qing Mu Xiang*	*Radix Aristolochiae*	Replaced by *Inulae Radix* since 2004
12	*Tong Di Jiao Nang (capsule)*	*Da Qing Mu Xiang*	*Aristolochia austroszechuanica*	Replaced by *Inulae Radixsince* since 2004
13	*Xiao Qing Long Tang (decoction)*	*Xi Xin*	*Asari radix et rhizoma*	Available
14	*Jiu Wei Qiang Huo Tang (decoction)*	*Xi Xin*	*Asari radix et rhizoma*	Available
15	*Dang Gui Si Ni Tang (decoction)*	*Xi Xin*	*Asari radix et rhizoma*	Available
16	*Ma Huang Fu Zi Xi Xin Tang (decoction)*	*Xi Xin*	*Asari radix et rhizoma*	Available

#	Drug names(In Mandarin Chinese)	AA and their derivatives (In Mandarin Chinese)	AA and their derivatives containing herbs	Status
17	*Ke Su Tan Chuan Wan (pill)*	*Ma Dou Ling*	*Fructus Aristolochiae*	Prescription Medicine
18	*Fu Fang She Dan Chuan Bei San(granule)*	*Ma Dou Ling*	*Fructus Aristolochiae*	Prescription Medicine
19	*Jing Zhi Ke Su Tan Chuan Wan (pill)*	*Ma Dou Ling*	*Fructus Aristolochiae*	Prescription Medicine
20	*Wei Fu Ke Li (granule)*	*Ma Dou Ling*	*Fructus Aristolochiae*	Prescription Medicine
21	*Zhi Ke Hua Tan Wan (pill)*	*Ma Dou Ling*	*Fructus Aristolochiae*	Prescription Medicine
22	*Chuan Xi Ling Jiao Nang (capsule)*	*Ma Dou Ling*	*Fructus Aristolochiae*	Prescription Medicine
23	*Fei An Pian (pill)*	*Ma Dou Ling*	*Fructus Aristolochiae*	Prescription Medicine
24	*Ji Ming Wan (pill)*	*Ma Dou Ling*	*Fructus Aristolochiae*	Prescription Medicine
25	*Ji Su Wan (pill)*	*Ma Dou Ling*	*Fructus Aristolochiae*	Prescription Medicine
26	*Qi Shi Wei Song Shi Wan (pill)*	*Ma Dou Ling*	*Fructus Aristolochiae*	Prescription Medicine
27	*Qing Guo Zhi Su Wan (pill)*	*Ma Dou Ling*	*Fructus Aristolochiae*	Prescription Medicine
28	*Run Fei Hua Tan Wan (pill)*	*Ma Dou Ling*	*Fructus Aristolochiae*	Prescription Medicine
29	*Shi San Wei Shu Gan Jiao Nang (capsule)*	*Ma Dou Ling*	*Fructus Aristolochiae*	Prescription Medicine
30	*Zhi Su Hua Tan Wan (pill)*	*Ma Dou Ling*	*Fructus Aristolochiae*	Prescription Medicine
31	*Xiao Ke Ping Chuan Kou Fu Ye (solution)*	*Ma Dou Ling*	*Fructus Aristolochiae*	Prescription Medicine
32	*Qing Guo Zhi Ke Wan (pill)*	*Ma Dou Ling*	*Fructus Aristolochiae*	Prescription Medicine
33	*Xin Bi Tao Xian Pian (pill)*	*Ma Dou Ling*	*Fructus Aristolochiae*	Prescription Medicine
34	*Zhi Ke Qing Guo Pian (pill)*	*Ma Dou Ling*	*Fructus Aristolochiae*	Prescription Medicine
35	*Zhi Su Hua Tan Jiao Nang (capsule)*	*Mi Ma Dou Ling*	*Fructus Aristolochiae*	Prescription Medicine
36	*Dou Shi Jiu Wei Neng Xiao San(granule)*	*Mu Xiang Ma Dou Ling*	*Aristolochia moupinens is*	Prescription Medicine
37	*Dou Shi Wu Wei Lv Rong Gao Jiao Nang (capsule)*	*Mu Xiang Ma Dou Ling*	*Aristolochia moupinens is*	Prescription Medicine
38	*Dou Shi Wu Wei Lv Rong Gao Wan (pill)*	*Mu Xiang Ma Dou Ling*	*Aristolochia moupinens is*	Prescription Medicine
39	*Dou Shi Wu Wei Song Shi Wan (pill)*	*Mu Xiang Ma Dou Ling*	*Aristolochia moupinens is*	Prescription Medicine
40	*Dou Shi Wu Wei Yu Gan Zi Wan (pill)*	*Mu Xiang Ma Dou Ling*	*Aristolochia moupinens is*	Prescription Medicine

#	Drug names(In Mandarin Chinese)	AA and their derivatives (In Mandarin Chinese)	AA and their derivatives containing herbs	Status
41	*Dou Shi Wu Wei Zhu Huang San (granule)*	*Mu Xiang Ma Dou Ling*	*Aristolochia moupinensis*	Prescription Medicine
42	*Feng Shi Sai Long Jiao Nang (capsule)*	*Mu Xiang Ma Dou Ling*	*Aristolochia moupinensis*	Prescription Medicine
43	*Feng Shi Zhi Tong Wan (pill)*	*Mu Xiang Ma Dou Ling*	*Aristolochia moupinensis*	Prescription Medicine
44	*Gan Chang Jiao Nang (capsule)*	*Mu Xiang Ma Dou Ling*	*Aristolochia moupinensis*	Prescription Medicine
45	*Jiu Wei Niu Huang Wan (pill)*	*Mu Xiang Ma Dou Ling*	*Aristolochia moupinensis*	Prescription Medicine
46	*Qi Wei Hong Hua Shu Sheng San (granule)*	*Mu Xiang Ma Dou Ling*	*Aristolochia moupinensis*	Prescription Medicine
47	*Qi Wei Hong Hua Shu Sheng Wan (pill)*	*Mu Xiang Ma Dou Ling*	*Aristolochia moupinensis*	Prescription Medicine
48	*Qing Fei Zhi Ke Wan (pill)*	*Mu Xiang Ma Dou Ling*	*Aristolochia moupinensis*	Prescription Medicine
49	*Si Wei Zhi Xie Mu Tang San (granule)*	*Mu Xiang Ma Dou Ling*	*Aristolochia moupinensis*	Prescription Medicine
50	*Wu Wei Zha Xun Wan (pill)*	*Mu Xiang Ma Dou Ling*	*Aristolochia moupinensis*	Prescription Medicine
51	*Tian Xian Teng San (granule)*	*Tian Xian Teng*	*Herba Aristolochiae*	Prescription Medicine
52	*He Wei Jiang Ni Jiao Nang (capsule)*	*Tian Xian Teng*	*Herba Aristolochiae*	Prescription Medicine
53	*Xiang Teng Jiao Nang (capsule)*	*Tian Xian Teng*	*Herba Aristolochiae*	Prescription Medicine
54	*Yun Xue Tang (decoction)*	*Tian Xian Teng*	*Herba Aristolochiae*	Prescription Medicine
55	*Fu Fang Feng Shi Yao Jiu (medicinal liquor)*	*Xun Gu Feng*	*herba Aristolochiae mollissimae*	Prescription Medicine
56	*Fu Fang Quan Can Pian (pill)*	*Xun Gu Feng*	*herba Aristolochiae mollissimae*	Prescription Medicine
57	*Qu Feng Chu Shi Yao Jiu (medicinal liquor)*	*Xun Gu Feng*	*herba Aristolochiae mollissimae*	Prescription Medicine
58	*San She Yao Jiu (medicinal liquor)*	*Xun Gu Feng*	*herba Aristolochiae mollissimae*	Prescription Medicine
59	*Shen Nong Yao Jiu (medicinal liquor)*	*Xun Gu Feng*	*herba Aristolochiae mollissimae*	Prescription Medicine
60	*Yi Shen Juan Bi Wan (pill)*	*Xun Gu Feng*	*herba Aristolochiae mollissimae*	Prescription Medicine
61	*Dou Zhong Zhuang Gu Jiao Nang (decoction)*	*Xun Gu Feng*	*herba Aristolochiae mollissimae*	Prescription Medicine
62	*Dou Zhong Zhuang Gu Wan (pill)*	*Xun Gu Feng*	*herba Aristolochiae mollissimae*	Prescription Medicine
63	*Feng Shi Ning Yao Jiu (medicinal liquor)*	*Xun Gu Feng*	*herba Aristolochiae mollissimae*	Prescription Medicine
64	*Shao Lin Zheng Gu Jing (pill)*	*Xun Gu Feng*	*herba Aristolochiae mollissimae*	Prescription Medicine

#	Drug names(In Mandarin Chinese)	AA and their derivatives (In Mandarin Chinese)	AA and their derivatives containing herbs	Status
65	*Yi Shen Juan Bi Wan (pill)*	*Xun Gu Feng*	*herba Aristolochiae mollissimae*	Prescription Medicine
66	*Fu Fang Wei Tong Jiao Nang (capsule)*	*Zhu Sha Lian*	*Aristolochia cinnabarina*	Prescription Medicine
67	*Jiu Long Jie Dou Jiao Nang (capsule)*	*Zhu Sha Lian*	*Aristolochia cinnabarina*	Prescription Medicine
68	*Bao Wei Jiao Nang (capsule)*	*Zhu Sha Lian*	*Aristolochia cinnabarina*	Prescription Medicine
69	*Jin Zhu Zhi Xie Pian (pill)*	*Zhu Sha Lian*	*Aristolochia cinnabarina*	Prescription Medicine
70	*Zhu Sha Lian Jiao Nang (capsule)*	*Zhu Sha Lian*	*Aristolochia cinnabarina*	Prescription Medicine

3. Supplementary files

Supplementary file 1. Detailed clinical characters of 90 UTUC patients.

Supplementary file 2. The output files of MutationalPatterns, including 90 UTUC, 26 UTUC cell-free DNA, eight matched primary tumors, two cell lines and three AA Sig subtype patients with matched normal tissue.

Supplementary file 3. The cosine similarities matrix.
